# A Novel Infrared and Visible Image Information Fusion Method Based on Phase Congruency and Image Entropy

**DOI:** 10.3390/e21121135

**Published:** 2019-11-21

**Authors:** Xinghua Huang, Guanqiu Qi, Hongyan Wei, Yi Chai, Jaesung Sim

**Affiliations:** 1 Key Laboratory of Complex System Safety and Control, Ministry of Education, Chongqing University, Chongqing 400044, China; huangxh1980@126.com (X.H.); weihy12@126.com (H.W.); chaiyi@cqu.edu.cn (Y.C.); 2 Computer Information Systems Department, Buffalo State College, Buffalo, NY 14222, USA; 3 College of Automation, Chongqing University of Posts and Telecommunications, Chongqing 400065, China; 4 Department of Mathematics and Computer Information Science, Mansfield University of Pennsylvania, Mansfield, PA 16933, USA; jsim@mansfield.edu

**Keywords:** image fusion, image entropy, PCNN, infrared and visible fusion, image decomposition, phase congruency

## Abstract

In multi-modality image fusion, source image decomposition, such as multi-scale transform (MST), is a necessary step and also widely used. However, when MST is directly used to decompose source images into high- and low-frequency components, the corresponding decomposed components are not precise enough for the following infrared-visible fusion operations. This paper proposes a non-subsampled contourlet transform (NSCT) based decomposition method for image fusion, by which source images are decomposed to obtain corresponding high- and low-frequency sub-bands. Unlike MST, the obtained high-frequency sub-bands have different decomposition layers, and each layer contains different information. In order to obtain a more informative fused high-frequency component, maximum absolute value and pulse coupled neural network (PCNN) fusion rules are applied to different sub-bands of high-frequency components. Activity measures, such as phase congruency (PC), local measure of sharpness change (LSCM), and local signal strength (LSS), are designed to enhance the detailed features of fused low-frequency components. The fused high- and low-frequency components are integrated to form a fused image. The experiment results show that the fused images obtained by the proposed method achieve good performance in clarity, contrast, and image information entropy.

## 1. Introduction

Both infrared and visible images are widely used in daily life. Due to the difference in wavelength, infrared and visible light contain different image information. Infrared images can reflect all the objects that emit infrared radiation. Visible-light images can provide the scene details. No matter whether an infrared or visible-light image, it is difficult for an image captured by a single shot to contain all-in-focus images in one scene. Infrared-visible fusion techniques can effectively combine the complementary information, which are the indicative features and detailed information extracted from infrared and visible images, respectively [1]. In the fused infrared-visible image, the targeted item can be highlighted and the corresponding indicative features as well as detailed information are retained. At present, image fusion techniques as a type of image pre-processing methods, especially for infrared-visible image fusion, have been widely applied to the target recognition in different environments, such as smart city, battlefield, remote sensing, and so on [2,3].

In recent years, transform domain based methods have become the mainstream in infrared-visible image fusion, which include pyramid, wavelet transform, multi-scale geometric transform, sparse representation [4,5], and so on. Pyramid, wavelet transform, and multi-scale geometric transform can be categorized as MST-based methods. MST-based methods have three main steps. First, MST is employed to decompose each source image into high-frequency sub-bands at different scales and directions as well as one low-frequency sub-band. Then, the obtained high- and low-frequency sub-bands are fused separately following different fusion rules. Finally, the fused image is obtained by performing the inverse MST on both fused high- and low-frequency sub-bands. Double-tree complex wavelet transform as a kind of wavelet transform can only capture a limited amount of edge information, but cannot correctly and effectively represent the discontinuity of lines and curves [6]. As a true two-dimensional (2D) multi-scale geometric analysis method, contourlet transform (CT) possesses localization, multi-resolution, multi-scale, multi-direction, and anisotropy.

As a shift-invariant version of CT, NSCT performs well in transform domain, and has been widely used in image fusion. NSCT has multi-scale and multi-direction features, which can solve the limitations of traditional wavelet methods in the representation of image curves and edges [7]. Compared with traditional MST-based image fusion methods, NSCT has shift invariance and also suppresses pseudo-Gibbs phenomenon [8,9]. Based on the above advantages, Liu proposed a general image fusion framework based on MST and sparse representation (SR) [10]. It overcomes the shortcomings of MST- and SR-based fusion methods at the same time. However, redundancy and loss of residue exist in this method [11]. Li proposed an infrared-visible image fusion method based on PC information, which fuses PC information into the coefficients of frequency bands [12]. However, the computational complexity of this fusion method is high.

In traditional infrared-visible images, the target information cannot be extracted effectively. Ding proposed an infrared-visible image fusion method based on non-downsampling shear transform (NSST) and sparse structure features [13]. First, source images are decomposed into high- and low-frequency sub-band coefficients. According to the advantages of principal component analysis (PCA) in principle information extraction, the PCA-based method is then used to fuse the low-frequency sub-band coefficients. At the same time, a new sparse-feature extraction method of high-frequency sub-band coefficients is proposed, which fuses the high-frequency components of source images. Finally, a fused image is obtained by inverse NSST. An infrared-visible image fusion method that integrates NSST and spiking cortical model was proposed by Kong [14]. This method uses NSST to reconstruct the decomposed components, which not only makes the fused image have good performance in human-eye visualization, but also effectively reduces the computational complexity. On the other hand, the fusion of different-scales and -direction sub-images can be realized by using a spiking cortical model. Xiang proposed an infrared-visible image fusion algorithm based on an adaptive dual-channel unit-linking PCNN in an NSCT domain [15]. This algorithm uses NSCT to decompose source images in multiple scales and directions. In order to make the adaptive dual-channel PCNN, the average gradient of each pixel is taken as the connection strength, and the time matrix adaptively determines the number of iterations. In the fusion process, a low-frequency sub-band and the modified spatial-frequency Laplacian of a high-frequency sub-band are used as input to excite the adaptive dual-channel unit-linking PCNN. Zhang proposed an NSCT-based infrared image fusion method, which used an adaptive Gaussian (AG) fuzzy membership method, compressed sensing (CS) technique, and total variation (TV) based gradient descent reconstruction algorithm to do the fusion calculation of infrared-visible image [8]. Wang proposed an infrared-visible image fusion method that integrates data compression based on sparse representation and compressed sensing [16]. This method first performs random projection compression on the remote sensing data, and then obtains the sparse coefficients of the compressed sample by sparse representation. Finally, fusion coefficients are combined with fusion influence factors, and the fused image is reconstructed by fusing sparse coefficients.

This paper proposes a novel precise decomposition framework for infrared-visible image fusion, in which image energy and details can be preserved well. First, NSCT is used to decompose source images to obtain corresponding high- and low-frequency components. The high-frequency sub-bands of each decomposed layer contain different information. For the top decomposed layer, the activity level of high-frequency coefficients is measured by a PCNN model [17]. For other decomposed layers, the absolute value of each high-frequency coefficient is taken as the activity level value following the absolute (ABS) maximum rule [10]. For low-frequency bands, PC is used as the image feature, whose value is not affected by image brightness, contrast, and illumination intensity. According to the information of PC, LSCM, and LSS, the low-frequency fusion rule is formulated. This rule enhances the detailed features of each source image. Finally, the fused image is reconstructed by performing inverse NSCT on the fused high- and low-frequency images. The main contributions of this paper can be summarized as follows:

- The high- and low-frequency components of source images are processed separately based on their own features.
- It applies PCNN and ABS to high-frequency sub-bands of different layers, which achieves a more precise decomposition of high-frequency components.
- The proposed image fusion algorithm can capture the details of source images well by integrating the advantages of NSCT, PCNN, and PC.

The rest of the sections of this paper are structured as follows: Section 2 proposes an infrared-visible image fusion framework based on an NSCT domain and specifies the corresponding technical details; Section 3 analyzes the results of comparative experiments; and Section 4 concludes this paper.

## 2. The Proposed Algorithm

**Figure 1:**
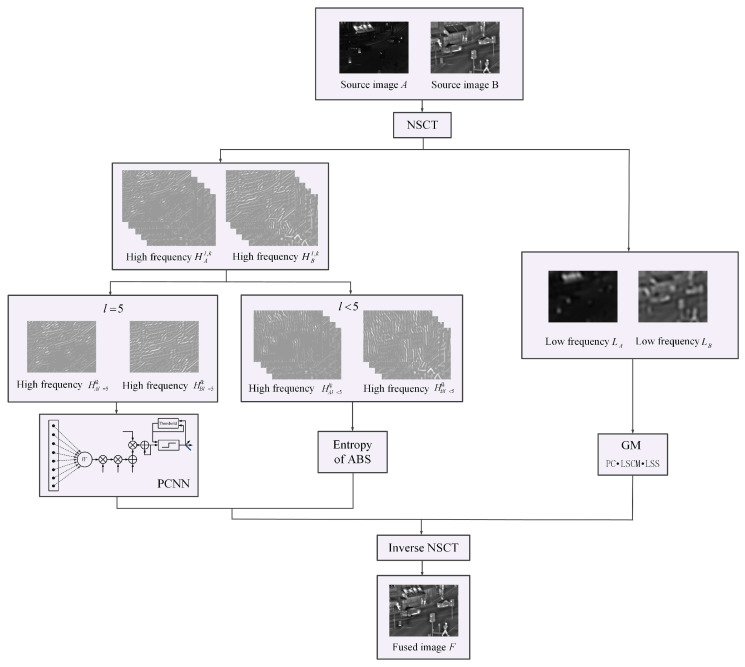
The proposed infrared-visible image fusion framework.

The proposed infrared-visible image fusion framework is shown in Figure 1, which has four main steps: image decomposition, the fusion of both high- and low-frequency sub-bands, and image reconstruction. It decomposes source images into 5-layer high- and low-frequency sub-bands first. Then, it applies different methods to the fusion of high- and low-frequency sub-bands, respectively. The decomposed high-frequency sub-bands are further categorized into two parts, $H_{A,l<5}^{l,k}$, $H_{B,l<5}^{l,k}$, and $H_{A,l=5}^{l,k}$, $H_{B,l=5}^{l,k}$. $H_{A,l<5}^{l,k}$ and $H_{B,l<5}^{l,k}$ are fused by the method of maximum absolute value. The fused high-frequency sub-bands contain the overall image structure information. PCNN is used to fuse $H_{A,l=5}^{l,k}$ and $H_{B,l=5}^{l,k}$. (The related details are explained in the following paragraph.) The fused low-frequency sub-bands retain the detailed information and the residual image information. Finally, it combines the fused high- and low-frequency sub-bands, which can make the fused image more informative.

### 2.1. NSCT

NSCT can overcome the frequency aliasing phenomenon caused by upsampling and downsampling on CT [18,19]. NSCT is a discrete image calculation framework that achieves shift-invariant, multi-scale, and multi-direction by using non-subsampled pyramid filter banks (NSPFBs) and non-subsampled directional filter banks (NSDFBs). Thus, the proposed solution uses NSCT to decompose source images into high- and low-frequency components.

Two source images are decomposed into high-frequency $\left\{H_{A}^{l,k},H_{B}^{l,k}\right\}$ and low-frequency $\left\{L_{A},L_{B}\right\}$ bands by performing *L*-level NSCT decomposition. $H_{A}^{l,k}$ and $H_{B}^{l,k}$ represent the high-frequency components at the decomposition level *l* and direction *k* of source image A and B, respectively, while $L_{A}$ and $L_{B}$ are the corresponding low-frequency components of source image A and B, respectively.

### 2.2. Fusion of High-Frequency Sub-Bands

The high-frequency sub-bands of different decomposed layers contain different information, which retains the overall image structure information. The maximum absolute value and PCNN fusion rules are applied to the fusion of different high-frequency sub-bands, which ensures that the structure information of source images is retained.

As shown in Equation (1), for the high-frequency sub-bands of the decomposed layer l=5, the activity level of high-frequency coefficients is measured by PCNN fusion rule. In our previous experiments, we used the different number of decomposition layers to test the performance of the proposed solution many times. According to the objective evaluation metrics, the corresponding results were compared. There is one trade-off between the performance and processing time. The performance of four decomposition layers is poor, and the processing time of six decomposition layers is long. Five decomposition layers can use a relatively short time to achieve a good performance in PCNN fusion. The proposed solution uses two different methods to fuse the high-frequency sub-bands from five decomposition layers. The method of maximum absolute value is used to fuse the high-frequency sub-bands from 1–4 layers. PCNN is applied to the fusion of the high-frequency sub-bands from the 5th layer. The fusion effects of the high-frequency sub-bands can be effectively improved, which is confirmed by the comparative experiments:

(1) $$H_{F}^{l,k}\left(i,j\right)=H_{F}^{l,k}{\left(i,j\right)}_{l=5}+H_{F}^{l,k}{\left(i,j\right)}_{l<5}.$$

In Equation (1), $H_{F}^{l,k}\left(i,j\right)$ represents the fused high-frequency coefficients. $H_{F}^{l,k}{\left(i,j\right)}_{l=5}$ represents the 5-level high-frequency fusion coefficients, which can be obtained by Equation (2). Equation (2) integrates the PCNN model, in which the entropy of the absolute value of high-frequency band is used as the network input. Then, the PCNN excitation times of high-frequency components $M_{A,ij}^{l,k}\left[N\right]$ and $M_{B,ij}^{l,k}\left[N\right]$ are calculated by Equation (3), where *N* denotes the number of iterations:

(2) $$H_{F}^{l,k}{\left(i,j\right)}_{l=5}=\left\{\begin{array}{c} H_{A}^{l,k}{\left(i,j\right)}_{l=5},\begin{array}{cc} if & M_{A,ij}^{l,k}\left[N\right]\geq M_{B,ij}^{l,k}\left[N\right], \end{array}\\ H_{B}^{l,k}{\left(i,j\right)}_{l=5},\begin{array}{cc} & otherwise, \end{array} \end{array}\right.$$

(3) $$M_{ij}\left[n\right]=M_{ij}\left[n-1\right]+P_{ij}\left[n\right],$$

where $P_{ij}\left[n\right]$ denotes the output model of PCNN [17].

**Figure 2:**
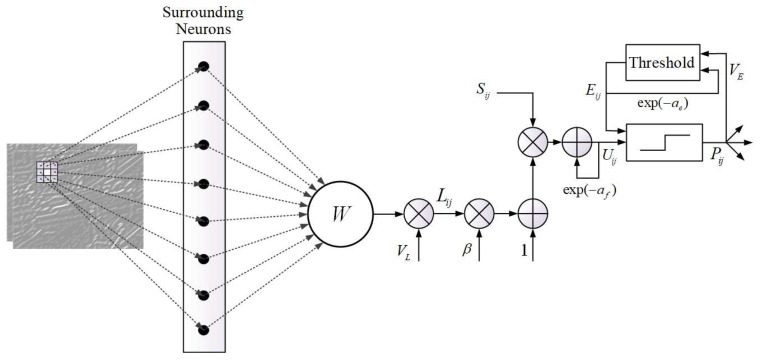
Architecture of the PCNN model used in the proposed method.

Figure 2 shows the architecture of PCNN model used in the proposed image fusion method. In PCNN, $F_{ij}\left[n\right]$ and $L_{ij}\left[n\right]$ are the feeding input and the linking input of the neuron at position (x,y) in iteration *n*, respectively, which can be obtained by Equations (4) and (5).

(4) $$F_{ij}\left[n\right]=S_{ij},$$

(5) $$L_{ij}\left[n\right]=V_{L}\sum_{kl}W_{ijkl}P_{kl}\left[n-1\right],$$

where $F_{ij}\left[n\right]$ is related to the intensity of input image $S_{ij}$ during the whole iteration process. $L_{ij}\left[n\right]$ is associated with the previous exciting status of eight surrounding neurons through the synaptic weights shown in Equation (6):

(6) $$W_{ijkl}=\left[\begin{array}{ccc} 0.5 & 1.0 & 0.5\\ 1.0 & 0.0 & 1.0\\ 0.5 & 1.0 & 0.5 \end{array}\right].$$

The parameter $V_{L}$ represents the amplitude of linking input. $U_{ij}\left[n\right]$ is the internal activity that consists of two terms, which can be calculated by Equation (7):

(7) $$U_{ij}\left[n\right]=e^{-a_{f}}U_{ij}\left[n-1\right]+F_{ij}\left[n\right]\left(1+\beta L_{ij}\left[n\right]\right).$$

In the first term, $e^{-a_{f}}U_{ij}\left[n-1\right]$ is a decay of its previous value, where the parameter $a_{f}$ is an exponential decay coefficient. The second term $F_{ij}\left[n\right]\left(1+\beta L_{ij}\left[n\right]\right)$ denotes the nonlinear modulation of $L_{ij}\left[n\right]$ and $F_{ij}\left[n\right]$, where the parameter β is the linking strength. The output module $P_{ij}\left[n\right]$ of the PCNN has two statuses, including excited ($P_{ij}\left[n\right]=1$) and unexcited ($P_{ij}\left[n\right]=0$):

(8) $$P_{ij}\left[n\right]=\left\{\begin{array}{c} 1,\begin{array}{cc} & \mathrm{if}\begin{array}{cc} & U_{ij}\left[n\right]>E_{ij}\left[n-1\right], \end{array} \end{array}\\ 0,\begin{array}{cc} & otherwise, \end{array} \end{array}\right.$$

(9) $$E_{ij}\left[n\right]=e^{-a_{e}}E_{ij}\left[n-1\right]+V_{E}P_{ij}\left[n\right].$$

The status depends on its two inputs, which are current internal activity $U_{ij}\left[n\right]$ and previous dynamic threshold $E_{ij}\left[n-1\right]$. According to Equation (9), the iteration is updating the dynamic threshold, where $a_{e}$ and $V_{E}$ are the exponential decay coefficient and the amplitude of $E_{ij}\left[n\right]$, respectively.

Similarly, $H_{F}^{l,k}{\left(i,j\right)}_{l<5}$ represents the 1-to-4 level high-frequency fusion coefficients, which can be obtained by Equation (10):

(10) $$H_{F}^{l,k}{\left(i,j\right)}_{l<5}=\left\{\begin{array}{ccc} H_{A}^{l,k}{\left(i,j\right)}_{l<5}, & & \begin{array}{c} Entropy(H_{A}^{l,k}{}_{l<5})\\ \geq Entropy(H_{B}^{l,k}{}_{l<5}), \end{array}\\ H_{B}^{l,k}{\left(i,j\right)}_{l<5}, & & otherwise. \end{array}\right.$$

In Equation (10), $Entropy({H_{A}^{l,k}}_{l<5})$ and $Entropy({H_{B}^{l,k}}_{l<5})$ represent the information entropy of high-frequency components $H_{A}^{l,k}$ and $H_{B}^{l,k}$, respectively. The information entropy of high frequency component $H_{x}^{l,k}$ can be calculated by Equation (11):

(11) $$Entropy\left(H_{x}^{l,k}\right)=\frac{1}{m\times n}\sum_{j=1}^{n}\sum_{i=1}^{m}\log_{2}\left|{H_{x}}^{l,k}\left(i,j\right)\right|,$$

where *m* and *n* are the total column and row number of $H_{x}^{l,k}$, and $|{H_{x}}^{l,k}\left(i,j\right)|$ is the maximum entropy of the ABS. The maximum entropy of the ABS is used as the fusion measurement of high-frequency sub-bands.

### 2.3. Fusion Rule of Low-Frequency Sub-Bands

The low-frequency sub-bands of NSCT filtered images mainly describe the detailed information that corresponds to the texture and edge information of source images. In medical imaging, organ or cell lesions are often identified by the detailed information. Thus, the enhancement of detailed features from each source image is the key of low-frequency sub-bands fusion.

This paper uses PC to enhance image features that make low-frequency sub-bands more informative. PC as a dimensionless measure can evaluate the significance of each image feature. In low-frequency sub-bands, PC value reflects the sharpness of image object. Thus, PC is used as the phase of the coefficient with maximal local sharpness. Since an image can be regarded as 2D signals [9], PC of an image pixel at location (x,y) can be calculated by Equation (12).

(12) $$PC\left(x,y\right)=\frac{\sum_{k}E_{\theta_{k}}\left(x,y\right)}{\varepsilon +\sum_{n}\sum_{k}A_{n,\theta_{k}}\left(x,y\right)}$$

where $\theta_{k}$ is the orientation angle at *k* scale [9], $A_{n,\theta_{k}}$ denotes the amplitude of the n-th Fourier component, and angle $\theta_{k}$, ε is a positive constant to remove the PC components of image signals. $E_{\theta_{k}}\left(x,y\right)$ can be calculated by Equation (13):

(13) $$E_{\theta_{k}}\left(x,y\right)=\sqrt{{F^{2}}_{\theta_{k}}\left(x,y\right)+{H^{2}}_{\theta_{k}}\left(x,y\right)},$$

where $F_{\theta_{k}}\left(x,y\right)=\sum_{n}b_{n,\theta_{k}}\left(x,y\right)$ and $H_{\theta_{k}}\left(x,y\right)=\sum_{n}c_{n,\theta_{k}}\left(x,y\right)$. $b_{n,\theta_{k}}\left(x,y\right)$ and $c_{n,\theta_{k}}\left(x,y\right)$ are the convolution results of input image pixel at location (x,y), which can be evaluated by Equation (14):

(14) $$\left[b_{n,\theta_{k}}\left(x,y\right),c_{n,\theta_{k}}\left(x,y\right)\right]=\left[I_{L}\left(x,y\right)*M_{n}^{b},I\left(x,y\right)*M_{n}^{c}\right],$$

where $I_{L}\left(x,y\right)$ is the low-frequency image pixel value at location (x,y). $M_{n}^{b}$ and $M_{n}^{c}$ are the even- and odd-symmetry filters of 2D log-Gabor at scale *n*. As a contrast invariant, PC has defects that do not reflect the local contrast changes. To compensate the lack of PC, a measure of sharpness change (SCM) shown in Equation (15) is developed:

(15) $$SCM\left(x,y\right)=\sum_{\left(x_{0},y_{0}\right)\in \Omega_{0}}{\left(I_{L}\left(x,y\right)-I_{L}\left(x_{0},y_{0}\right)\right)}^{2},$$

where $\Omega_{0}$ represents a local area at location (x,y). Meanwhile, LSCM shown in Equation (16) is introduced to calculate the contrast of location (x,y) neighborhood:

(16) $$LSCM\left(x,y\right)=\sum_{i=-M}^{M}\sum_{j=-N}^{N}SCM\left(x+i,y+j\right),$$

where $\left(2M+1\right)\times \left(2N+1\right)$ denotes the neighborhood size. Since LSCM and PC cannot fully reflect the local signal strength, LSS shown in Equation (17) is introduced:

(17) $$LSS\left(x,y\right)=\underset{i\in (-M,M)}{Max}\underset{j\in (-N,N)}{Max}\left|x_{ij}-\mu_{MN}\right|,$$

where $x_{ij}$ is the pixel in location of this image patch, $\mu_{MN}$ represents the mean value of this image patch.

As shown in Equation (18), a global measurement (GM) is proposed that integrates PC, LSCM, and LSS complements to measure different aspects of image information:

(18) $$GM\left(x,y\right)={\left(PC\left(x,y\right)\right)}^{\alpha }\cdot {\left(LSCM\left(x,y\right)\right)}^{\beta }\cdot {\left(LSS\left(x,y\right)\right)}^{\gamma },$$

where α, β, and γ are the parameters used in GM to adjust PC, LSCM, and LSS, respectively. When GM is obtained, the fused image of low-frequency sub-bands can be calculated by the rule proposed in Equation (19):

(19) $$L_{F}\left(x,y\right)=\left\{\begin{array}{cc} L_{A}\left(x,y\right), & if\;Lmap_{A}\left(x,y\right)=1,\\ L_{B}\left(x,y\right), & otherwise, \end{array}\right.$$

where $L_{F}\left(x,y\right)$, $L_{A}\left(x,y\right)$, $L_{B}\left(x,y\right)$ are low-frequency sub-bands of the fused image, source image $I_{A}$ and $I_{B}$, respectively. $Lmap_{i}\left(x,y\right)$ denotes a decision map for the fusion of low-frequency sub-bands, which can be calculated by Equation (20):

(20) $$Lmap_{i}\left(x,y\right)=\left\{\begin{array}{cc} 1, & if\left\lceil \Phi_{i}\left(x,y\right)\right\rceil >\frac{\tilde{M}\times \tilde{N}}{2},\\ 0, & otherwise, \end{array}\right.$$

where ⌈⌉ is the cardinality of a set, and $\Phi_{i}\left(x,y\right)$ can be calculated by Equation (21). The cardinality of a set is helpful to obtain the abundant image details and structure information:

(21) $$\Phi_{i}\left(x,y\right)=\left\{\begin{array}{c} \left(x_{0},y_{0}\right)\in \Omega_{1}|GM_{i}\left(x_{0},y_{0}\right)\geq \\ \max(GM_{1}\left(x_{0},y_{0}\right),...,GM_{i-1}\left(x_{0},y_{0}\right),\\ GM_{i+1}\left(x_{0},y_{0}\right),...,GM_{K}\left(x_{0},y_{0}\right)) \end{array}\right\}$$

where $\Omega_{1}$ represents a sliding window with a size of $\tilde{M}\times \tilde{N}$ centered at location (x,y), and *K* is the number of source images. GM defined in Equation (18) is expressed as a general term. In Equation (21), the subscript of GM is used to select the corresponding maximum value from source images.

For input source images A and B, the high-frequency components $\left\{H_{A}^{l,k},H_{B}^{l,k}\right\}$ and low-frequency components $\left\{L_{A},L_{B}\right\}$ are first obtained by NSCT decomposition. The activity level of high-frequency components $\left\{H_{A}^{l,k},H_{B}^{l,k}\right\}$ is then measured by using the absolute maximum rule and PCNN model. Meanwhile, it applies PC to the fusion of low-frequency components. Finally, the fused high- and low-frequency components $H_{F}$ and $L_{F}$ are inversely transformed by NSCT to obtain the fused image $I_{F}$. Algorithm 1 shows the main steps of the proposed infrared-visible image fusion solution.

**Algorithm 1** The proposed infrared-visible image fusion algorithm

**Input:**
 source image A and B Parameters: decomposition layer *l*, decomposition direction *k*


**Input:**
 fused image F

1:**for** each source image A and B **do**2:Decompose source image A and B into corresponding high- and low-frequency sub-bands $\left\{H_{A}^{l,k},H_{B}^{l,k}\right\}$ and $\left\{L_{A},L_{B}\right\}$ by NSCT respectively3:
**end for**
4:**for** each decomposed layer **do**5: **if** the decomposed layer l=5 of high-frequency sub-bands **then**6:  Measure the activity level of high-frequency coefficients by PCNN.7:  Obtain the $5^{th}$ layer fusion coefficient of high-frequency sub-bands by PCNN as follows:  $H_{F}^{l,k}{\left(i,j\right)}_{l=5}=\left\{\begin{array}{c} H_{A}^{l,k}{\left(i,j\right)}_{l=5},\;if\;M_{A,ij}^{l,k}\left[N\right]\geq M_{B,ij}^{l,k}\left[N\right]\\ H_{B}^{l,k}{\left(i,j\right)}_{l=5},\;otherwise \end{array}\right.$
8: **end if**
9: **if** the decomposed layer l<5 of high-frequency sub-bands **then**10:  Use the maximum entropy of the ABS of coefficient as the actually measured value of activity level.11:  Obtain the first four-layer coefficients of high-frequency sub-bands as follows:  $H_{F}^{l,k}{\left(i,j\right)}_{l<5}=\left\{\begin{array}{cc} H_{A}^{l,k}{\left(i,j\right)}_{l<5}, & \;\begin{array}{c} Entropy\left(H_{A}^{l,k}{}_{l<5}\right)\\ \geq Entropy\left(H_{B}^{l,k}{}_{l<5}\right) \end{array}\\ H_{B}^{l,k}{\left(i,j\right)}_{l<5}, & \;\;otherwise \end{array}\right.$
12: **end if**13: The obtained image of high-frequency sub-bands is $H_{F}^{l,k}\left(i,j\right)=H_{F}^{l,k}{\left(i,j\right)}_{l=5}+H_{F}^{l,k}{\left(i,j\right)}_{l<5}$14:
**end for**
15:**for** each source image A and B **do**16:It uses PC, LSCM, and LSS to design GM: $GM\left(x,y\right)={\left(PC\left(x,y\right)\right)}^{\alpha }\cdot {\left(LSCM\left(x,y\right)\right)}^{\beta }\cdot {\left(LSS\left(x,y\right)\right)}^{\gamma }$
17: Calculate the image of low-frequency sub-bands by the following rule: $L_{F}\left(x,y\right)=\left\{\begin{array}{c} L_{A}\left(x,y\right)\;if\;Lmap_{A}\left(x,y\right)=1\\ L_{B}\left(x,y\right)\;\;\;otherwise \end{array}\right.$
18:
**end for**
19:The inverse NSCT is applied to the obtained images of high- and low-frequency sub-bands $\left\{H_{F},L_{F}\right\}$ to get the fused image *F*.


## 3. Comparative Experiments

### 3.1. Experiment Preparation

In comparative experiments, 30 sets of infrared-visible images are used to test the fusion performance. The resolution of test images are 256∗256. The infrared wavelength is 700–2526 nm, and the visible wavelength is 390–700 nm. Infrared-visible image pairs were collected by Liu [10] and can be downloaded from quxiaobo.org. All the experiment’s program’s codes are programmed in Matlab 2014a (MathWorks, Natick, MA, USA) on an Intel(R) Core(TM)i7-4790CPU (Intel, Santa Clara, CA, USA) @ 3.60 GHz Desktop with 8.00 GB RAM.

### 3.2. Objective Evaluation Metrics

For the evaluation of fused image, a single evaluation metric cannot fully reflect the performance of fused image [20,21]. Therefore, it is necessary to use multiple metrics to do comprehensive performance analysis. This paper uses five objective metrics to evaluate the performances of different fusion methods, which include $Q^{TE}$[22,23], $Q^{AB/F}$[24,25], $Q^{MI}$[23], $Q^{CB}$[23,26], and $Q^{VIF}$ [25,27]. $Q^{TE}$ is used to evaluate the Tsallis entropy of the fused image. $Q^{AB/F}$ as a gradient-based quality index measures the edge information. $Q^{MI}$ is used to evaluate the similarity between the fused image and source images. Both $Q^{CB}$ and $Q^{VIF}$ measure the human visual performance of the fused image.

### 3.3. Experiment Results of Infrared-Visible Image Fusion

In this section, the proposed NSCT-based fusion framework is compared with seven popular fusion methods, such as the adaptive spare representation (ASR) based image fusion method proposed by Liu [28], the convolutional neural network (CNN) based image fusion method proposed by Liu [29], the multi-channel medical image fusion (CT) proposed by Zhu [25], the multi-modality image fusion method with joint patch clustering based dictionary learning (KIM) proposed by Kim [30], the image fusion based on multi-scale transform and sparse representation (MST-SR) proposed by Liu [10], a novel infrared and visual image fusion algorithm based on NSST and improved PCNN (NSST-PCNN) was proposed by Li [31], and an infrared and visible image fusion scheme based on NSCT and PC information (NSCT-PC) proposed by Li [9]. This section only picks the fused results of six comparative experiments from thirty attempts to analyze the fusion performance.

#### 3.3.1. Comparative Experiments—1

**Figure 3:**
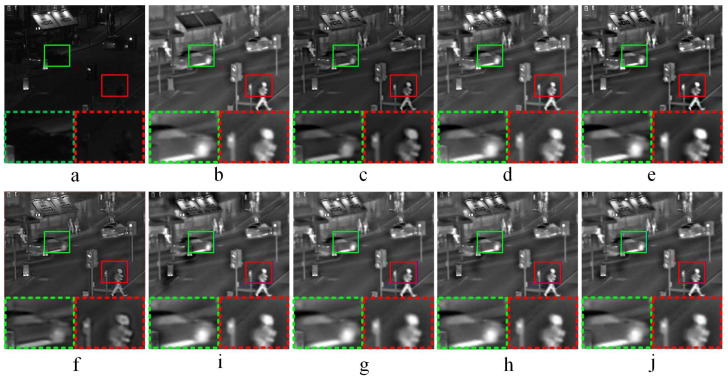
Infrared-visible image fusion comparative experiments—1. (**a**,**b**) are source images, (**c**–**j**) are the fused results of ASR, CNN, CT, KIM, MST-SR, NSCT-PCNN, NSCT-PC, and the proposed method, respectively. At the bottom of each image, two areas marked in green and red dashed line frames correspond to the magnified areas encompassed in green and red frames, respectively.

Figure 3 shows the fused results of infrared-visible image fusion experiment—1. As shown in Figure 3c,f, the fused images obtained by ASR and KIM have low brightness. The light brightness in source image (a) is not well preserved in both (c) and (f), so images (c) and (f) have overall poor visual performance. The CNN method does not perform well in some local areas as shown in Figure 3d. According to the partially enlarged areas in Figure 3e, some local areas of the fused image obtained by CT have high brightness, and the image detailed information is not obvious. In Figure 3i, the saturation of the fused image is high, and the edge detailed information is not obvious. In addition, the fused image obtained by NSCT-PCNN has low contrast, and the global image features have poor performance. Compared the fused images (h) and (j) as well as the corresponding partially magnified images in Figure 3, NSCT-PC and the proposed method have the close visual performance of human eyes.

#### 3.3.2. Comparative Experiments—2

**Figure 4:**
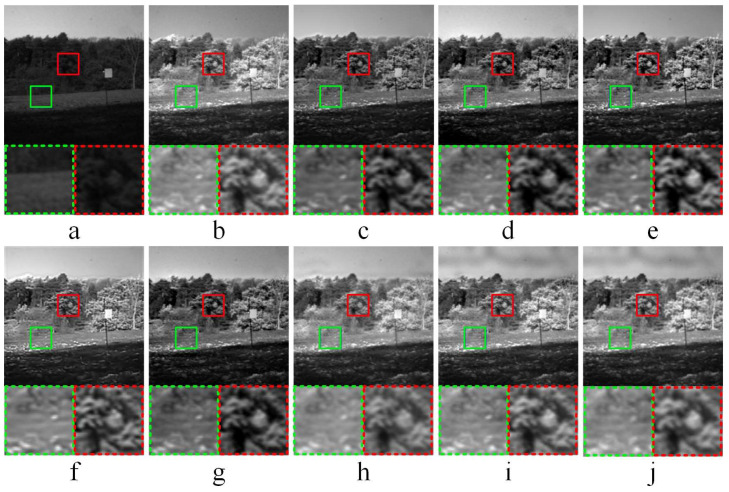
Infrared-visible image fusion comparative experiments—2. (**a**,**b**) are source images, (**c**–**j**) are the fused results of ASR, CNN, CT, KIM, MST-SR, NSCT-PCNN, NSCT-PC, and the proposed method, respectively. At the bottom of each image, two areas marked in green and red dashed line frames correspond to the magnified areas encompassed in green and red frames, respectively.

Figure 4 shows the fused results of infrared-visible image fusion experiment—2. After the comparisons of fused images obtained by different methods, it gets the following conclusions. In Figure 4c,f, the fused images obtained by ASR and KIM have low brightness, and poor performance in global features. As shown in the magnified areas of Figure 4d,e, CNN does not obtain the clear details of fused image, the contrast of the partially enlarged image obtained by CT is high, and the corresponding edge information is not obvious. For the fused image (f) in Figure 4 obtained by KIM, the connection area of sky and forest has high edge brightness. As shown in Figure 4h, the fused image obtained by NSCT-PCNN has high brightness and poor visual effect. Compared with the experiment results of the other six fusion methods, the fused images obtained by NSCT-PC and the proposed method have better fusion performance.

#### 3.3.3. Comparative Experiments—3

**Figure 5:**
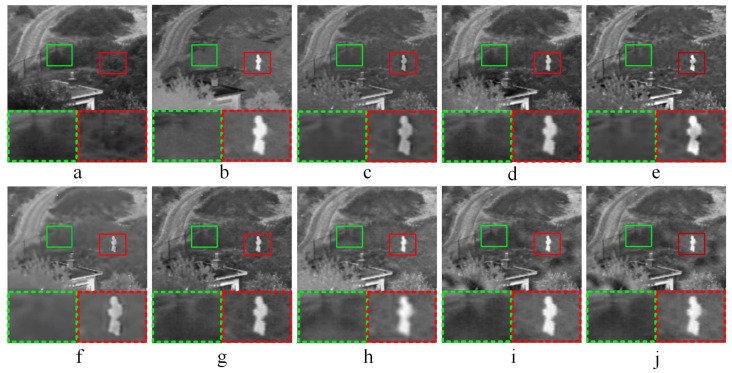
Infrared-visible image fusion comparative experiments—3. (**a**,**b**) are source images, (**c**–**j**) are the fused results of ASR, CNN, CT, KIM, MST-SR, NSCT-PCNN, NSCT-PCand the proposed method, respectively. At the bottom of each image, two areas marked in green and red dashed line frames correspond to the magnified areas encompassed in green and red frames, respectively.

Figure 5 shows the fused results of infrared-visible image fusion experiment—3. In Figure 5c,f, both ASR and KIM obtain the fused images with high brightness, and do not preserve the detailed information of source image (b). Comparing with ASR, the fused image obtained by KIM is fuzzy and not conducive to human-eye observation. As shown in Figure 5d, the fused image obtained by CNN has high saturation. In Figure 5e,g, the detail texture information of fused images obtained by CT and MST-SR is not clear by observing the partially enlarged areas. Compared with the proposed method, the fused image obtained by NSCT-PCNN in Figure 5h has low saturation and poor performance in global features. As shown in Figure 5i,j, NSCT-PC and the proposed method have good performance in both global and local features.

#### 3.3.4. Comparative Experiments—4

**Figure 6:**
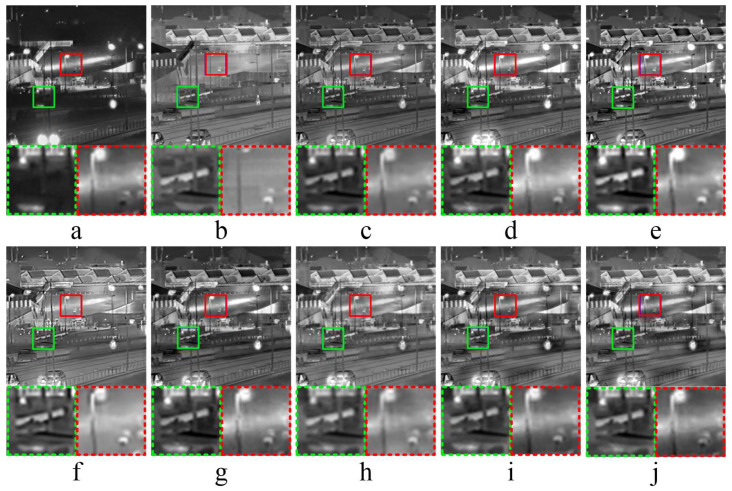
Infrared-visible Image Fusion Comparative Experiments—4. (**a**,**b**) are source images, (**c**–**j**) are the fused results of ASR, CNN, CT, KIM, MST-SR, NSCT-PCNN, NSCT-PCand the proposed method, respectively. At the bottom of each image, two areas marked in green and red dashed line frames correspond to the magnified areas encompassed in green and red frames, respectively.

Figure 6 shows the fused results of infrared-visible image fusion experiment—4. In Figure 6c, the fused image obtained by ASR has a general visualization performance. As shown in Figure 6d, the car light has high brightness in the fused image obtained by CNN. In Figure 6e,g, the fused images obtained by CT and MST-SR are dark, and have poor overall visualization performance. As shown in Figure 6f,h, the fused images obtained by KIM and NSCT-PC have high brightness. After the analysis of detailed information, the detailed textures of fused images are not obvious, which are not conducive to human-eye observation. Comparing with NSCT-PC, the proposed method has better performance in both global and local features of source images.

#### 3.3.5. Analysis of Comparative Experiment Results

**Figure 7:**
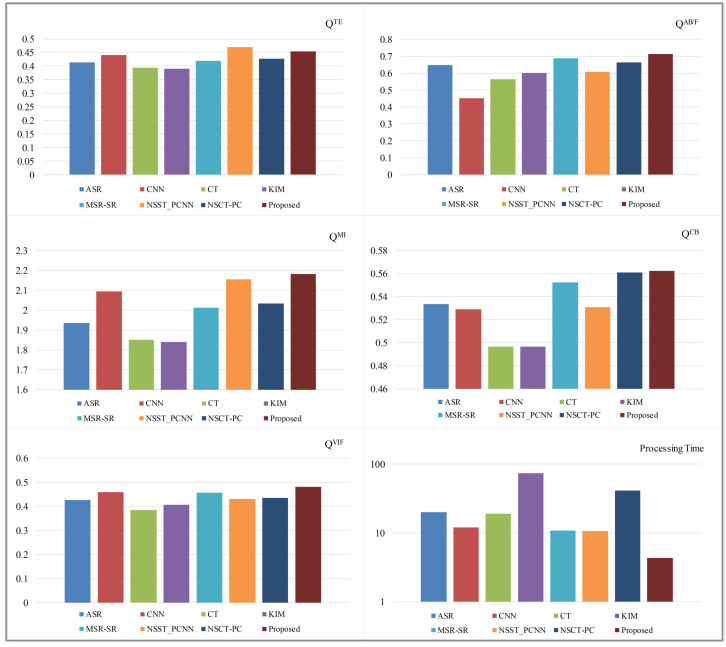
Average objective evaluations of thirty infrared-visible image fusion comparative experiments.

As the analysis of 30 comparative experiments, Table 1 and Figure 7 show the average objective evaluation results of infrared-visible image fusion. In Table 1, all the best results are marked in bold. According to the results shown in Table 1 and Figure 7, the proposed method achieves the best performance in $Q^{AB/F}$, $Q^{MI}$, $Q^{CB}$, and $Q^{VIF}$, and the second best performance in $Q^{TE}$. $Q^{TE}$ of the proposed method is a little bit lower than the best one obtained by NSST-PCNN. It means that both the proposed method and NSST-PCNN can retain more information of source images. Meanwhile, the similarities between the fused images obtained by these two methods and source images are also comparable. For the $Q^{AB/F}$ metric, the proposed method is slightly higher than other methods. Thus, the proposed method performs better in the preservation of image edge details. Additionally, the proposed method can also preserve the global and local features of source images well, and also achieve a good performance in human-eye visualization. As shown in Figure 7, the proposed method uses the shortest processing time in infrared-visible image fusion among all the eight fusion methods, which is much less than others as well as about 40% of the second shortest processing time. Thus, the results of comparative experiments confirm that the proposed infrared-visible image fusion solution has a low algorithm complexity and can effectively reduce the related costs.

## 4. Conclusions

In this paper, an NSCT-based precise high-frequency decomposition method for infrared-visible image fusion is proposed. The fusion method combines NSCT, PCNN model, and PC information to improve the visual quality of fused images. Specifically, the method uses NSCT to achieve the high- and low-frequency decomposition of source images. The fusion of high-frequency image coefficients is realized by introducing PCNN and ABS as the activity metrics of high-frequency coefficients. In the fusion of low-frequency components, it integrates the fusion rules of LSCM, LSS, and PC features to achieve the energy preservation and detail extraction of low-frequency components. Finally, the fused image is obtained by inverse NSCT over the fused high- and low-frequency components. Compared to other image fusion methods, the proposed method achieves good performance on the structural similarity and detail preservation in fused images. The experiment results confirm that the proposed method has good effectiveness and high speed in infrared-visible image fusion.

In the future, the proposed method will be optimized to increase the processing speed. A weighted fusion will be explored to improve the fusion performance. The statistical tests, such as Friedman’s test, will be introduced to compare the performance of the proposed method. The proposed image fusion method will also be extended to other multi-modality image fusion areas, such as medical image fusion, multi-focus image fusion, and so on as well as face recognition, especially in night scenes. 

## Figures and Tables

**Table 1:** Average objective evaluations of thirty infrared-visible image fusion comparative experiments.

	$Q^{TE}$	$Q^{AB/F}$	$Q^{MI}$	$Q^{CB}$	$Q^{VIF}$
ASR	0.4123	0.6470	1.9354	0.5334	0.4250
CNN	0.4402	0.4528	2.0952	0.5288	0.4582
CT	0.3931	0.5639	1.8511	0.4965	0.3848
KIM	0.3896	0.6011	1.8408	0.4966	0.4062
MSR-SR	0.4195	0.6888	2.0132	0.5524	0.4563
NSST-PCNN	**0.4697**	0.6082	2.1546	0.5308	0.4299
NSCT-PC	0.4262	0.6639	2.0337	0.5608	0.4352
Proposed	0.4541	**0.7122**	**2.1813**	**0.5622**	**0.4811**
